# A protective bivalent vaccine against Rift Valley fever and bluetongue

**DOI:** 10.1038/s41541-020-00218-y

**Published:** 2020-07-30

**Authors:** Eva Calvo-Pinilla, Alejandro Marín-López, Sandra Moreno, Gema Lorenzo, Sergio Utrilla-Trigo, Luis Jiménez-Cabello, Julio Benavides, Aitor Nogales, Rafael Blasco, Alejandro Brun, Javier Ortego

**Affiliations:** 1 Instituto Nacional de Investigación y Tecnología Agraria y Alimentaria, Centro de Investigación en Sanidad Animal (INIA-CISA), Madrid, Spain; 2 Section of Infectious Diseases, Department of Internal Medicine, Yale University School of Medicine, New Haven, CT USA; 3 Instituto de Ganadería de Montaña (CSIC-Universidad de León), León, Spain; 4 Instituto Nacional de Investigación y Tecnología Agraria y Alimentaria, Departamento de Biotecnología, Madrid, Spain

**Keywords:** Biotechnology, Immunology, Microbiology

## Abstract

Rift Valley fever (RVF) and bluetongue (BT) are two important ruminant diseases transmitted by arthropods. Both viruses have shown important geographic spread leading to endemicity of BT virus (BTV) in Africa and Europe. In this work, we report a dual vaccine that simultaneously induces protective immune responses against BTV and RVFV based on modified vaccinia Ankara virus (MVA) expressing BTV proteins VP2, NS1, or a truncated form of NS1 (NS1-Nt), and RVFV Gn and Gc glycoproteins. IFNAR^(−/−)^ mice immunized with two doses of MVA-GnGc-VP2 developed a significant neutralizing antibody response against BTV-4 and RVFV. Furthermore, the homologous prime-boost immunization with MVA-GnGc-NS1 or MVA-GnGc-NS1-Nt triggered neutralizing antibodies against RVFV and NS1-specific cytotoxic CD8+ T cells in mice. Moreover, all mice immunized with MVA-GnGc-NS1 or MVA-GnGc-NS1-Nt remained healthy after lethal challenge with RVFV or BTV-4. The homologous prime-boost vaccination with MVA-GnGc-NS1, which was the best immunization strategy observed in mice, was assayed in sheep. Clinical signs and viremia were absent or highly reduced in vaccinated sheep after challenge with BTV-4 or RVFV. These results indicate that MVA-GnGc-NS1 vaccination elicits immune protection against RVFV and BTV in sheep.

## Introduction

Ruminants are affected by a variety of viral infections, including Rift Valley fever virus (RVFV) and bluetongue virus (BTV). These pathogens cause epidemics of severe disease, particularly in sheep, with serious implications for agricultural livestock and trade. BTV is globally distributed, being the Antarctica the only continent free of BTV infection for now^1^. Although RVFV is mainly present in Africa, recent outbreaks in the Middle East have demonstrated its potential to spread beyond the African continent^2^. As vector-borne diseases, these viruses can be transmitted very rapidly through several species of hematophagous insects^3,4^; which, coupled with global climate change increase the risk of introduction of these viruses in non-endemic regions by the expansion of the vector’s range. In order to prevent the spread of these arboviruses, improved vaccination strategies need to be developed. The use of multivalent vaccines that could provide immunity against a prevalent disease for which vaccination is mandatory, i.e., BTV in Europe, while immunizing against other diseases of more sporadic nature, such as RVFV would be a potential strategy to overcome this problem and to reduce the cost of vaccine production.

**Fig. 1:**
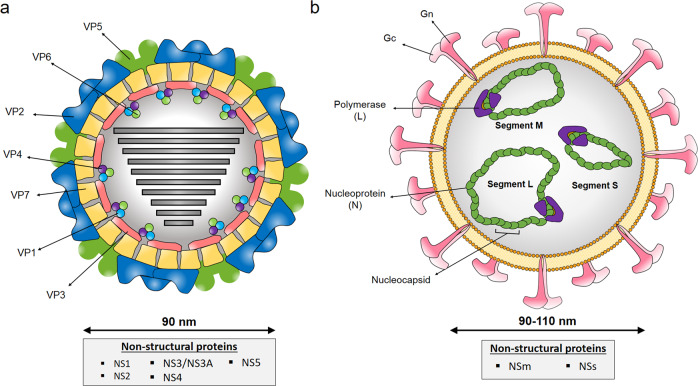
Diagrammatic representation of the viral particles of BTV and RVFV. **a** Three concentric layers constituted by VP2 and VP5 (outer capsid), VP7 (intermediate layer), and VP3 (subcore) characterized BTV virions (~90 nm in diameter). The RNA polymerase complex, which is located inside the inner capsid, is composed by structural proteins VP1, VP4, and VP6. Five additional proteins (NS1, NS2, NS3/NS3A, NS4, and NS5) are synthesized inside the cell during the replicative cycle. VP2 and NS1 proteins of BTV are expressed by the recombinant MVAs. **b** Enveloped virions of RVFV (~90–110 nm in diameter) are characterized by a negative or ambisense RNA genome composed of three single-stranded segments (designated L, M, and S). These three RNA molecules are encapsidated by the nucleoprotein (N), shaping the nucleocapsid which interacts with the viral polymerase (L). Glycoproteins Gn and Gc, expressed by recombinant MVAs, elicit production of virus-neutralizing antibodies. Nonstructural proteins NSm and NSs are expressed during infection.

Although live-attenuated RVF vaccines are used to control the disease in Africa, they have major safety concerns^5^. RVFV is a negative single-stranded RNA virus belonging to the *Bunyavirales* order and family *Phenuviridae* that occurs as a single serotype. The RVFV genome is composed of three segments, large (L), medium (M), and small (S; Fig. 1). The M segment encodes two glycoproteins Gn and Gc, involved in cell attachment and virus–cell membrane fusion, and two accessory proteins^6^. As Gn and Gc are the major antigenic components on the viral membrane and are the main inducers of neutralizing antibodies^7^, they are ideal targets for vaccine development. These glycosylated proteins have been also shown to stimulate a robust T-cell response correlated with protective immunity against virus infection^8–10^.

BTV belongs to the *Reoviridae* family, genus *Orbivirus*, and has a segmented dsRNA genome^11^. This virus has undergone considerable expansion worldwide over the past decades with 27 serotypes described to date^12^. Control of BT mainly relies in the use of inactivated vaccines against prevalent BTV; however, these do not offer cross-protection among serotypes. Antigenic variability of BTV is the major obstacle of cross-protective immunity. VP2 protein forms, together with VP5, the outer capsid of BTV particle (Fig. 1), and is involved in cell attachment and virus entry^11,13^. This protein is the main target of neutralizing antibodies^14^. Although neutralizing antibodies directed to BTV VP2 protein can prevent infection with homologous BTV, these are serotype specific. Thus, vaccines that induce a cross-reactive T-cell response are needed to elicit multiserotype protection. NS1 is the most synthesized viral protein in BTV-infected cells and is involved in upregulation of viral protein synthesis^15^. This protein contains epitopes associated with both T-cell and humoral responses^16,17^. Importantly, the amino acid sequence of BTV NS1 protein is highly conserved between BTV serotypes, and we have previously shown that the cellular immune response against NS1 or its N-terminal region (NS1-Nt) is protective against heterologous serotypes^18,19^.

In previous works, BTV NS1 and VP2 proteins and RVFV Gn/Gc proteins were described as promising candidate antigens for the development of recombinant vaccines. Recombinant MVAs expressing those proteins were able to provide efficient protection against BTV or RVFV virulent challenge in mice^19–21^. In this work, MVA-vectored vaccines were designed to express these highly immunogenic antigens of BTV and RVFV simultaneously. We analyzed the adaptive immune responses and protection of these vaccines in both mice and sheep, and we showed that MVA-GnGc-NS1 conferred protection against BTV or RVFV challenges in both, the murine model and the natural host.

## Results

### Expression of heterologous proteins by MVA vectors

**Fig. 2:**
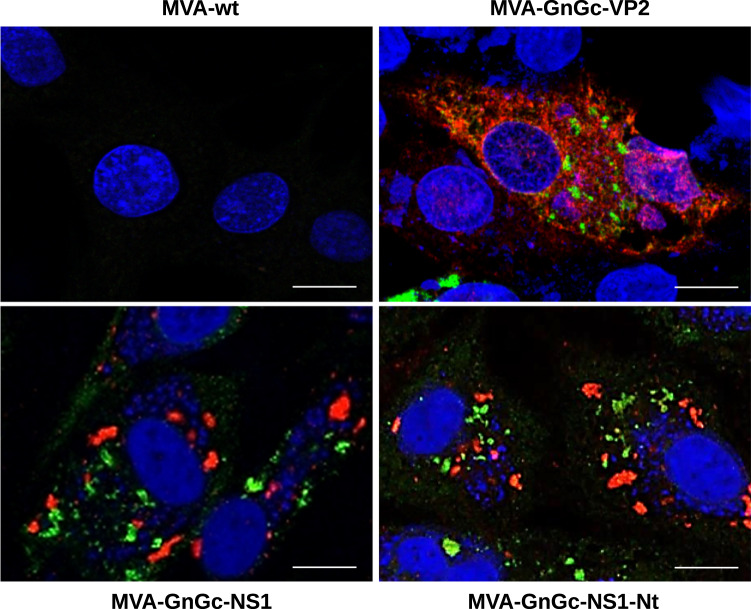
Expression analysis of heterologous proteins by MVA-GnGc-VP2, MVA-GnGc-NS1, and MVA-GnGc-NS1-Nt vectors. DF-1 cells were infected with recombinant MVAs (MOI 0.1) and immunofluorescence analysis was performed at 48 h.p.i. Expression of Gn and Gc was detected by staining with serum from sheep infected with RVFV and a secondary green antibody. BTV proteins (VP2, NS1, and NS1-Nt) were detected with BTV-infected mouse serum and a secondary red antibody. Images visualized by confocal microscopy (63×). Scale bars 10 µm.

Recombinant dual MVA viruses expressing the RVFV Gn and Gc glycoproteins, and BTV proteins VP2, NS1, or a truncated form of NS1 (NS1-Nt) were generated. A RVFV GnGc polyprotein sequence was inserted into the F13L locus, while the BTV segments were cloned into the TK locus, generating the MVA-GnGc-VP2, MVA-GnGc-NS1, and MVA-GnGc-NS1-Nt constructs as described in “Methods” section. To determine whether the heterologous BTV and RVFV proteins were efficiently expressed by the dual MVA system, DF-1 cells were infected with each recombinant virus for 48 h and immunofluorescence assays were carried out. Figure 2 confirms the efficient expression of RVFV glycoprotein antigens, and BTV VP2, NS1, and NS1-Nt proteins in cells infected with the dual MVA vaccine vectors that were used for immunization subsequently.

### Immune responses induced by dual MVA against proteins of RVFV and BTV in mice

A number of experimental vaccines for BTV and RVFV have been previously studied in the mouse model based on IFNAR^(−/−)^ mice^22–28^. Although extrapolation of findings in mice to natural hosts must be done with care due to differences in the biology between mouse and ruminants, experimental infections of IFNAR^(−/−)^ mice with several studied arboviruses, such as BTV and RVFV closely mimics hallmarks of these viruses in their natural host^29^.

**Fig. 3:**
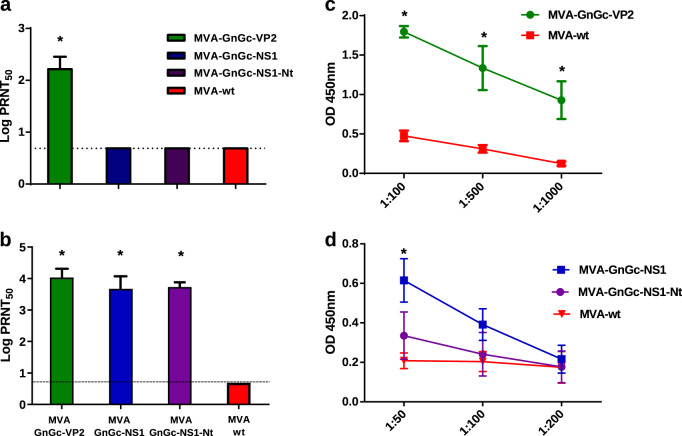
Humoral responses against BTV and RVFV elicited by MVA recombinant vaccines 2 weeks post booster in mice. Induction of virus-neutralizing antibodies against BTV **a** and RVFV **b** in animals immunized with MVA recombinant vaccines by plaque reduction neutralization assay. The columns represent the mean of the group and error bars that indicate the SD. Cutoff: 0.69 (log 5). **c** Induction of IgG VP2 antibodies by indirect ELISA in MVA-GnGc-VP2-vaccinated animals. Three dilutions of sera were tested (*x*-axis) and lines represent media. **d** Induction of IgG NS1 antibodies by indirect ELISA in MVA-GnGc-NS1- and MVA-GnGc-NS1-Nt-vaccinated mice. Three dilutions of sera were tested (*x*-axis). Lines represent means and error bars the SD. Statistical differences were calculated by Mann–Whitney nonparametric tests; *p ≤ 0.05.

IFNAR^(−/−)^ mice were immunized twice with MVA-GnGc-NS1, MVA-GnGc-NS1-Nt, MVA-GnGc-VP2, or MVA-wt (mock-vaccinated control), and humoral and cellular immune responses were measured 14 or 10 days after boost, respectively. MVA-GnGc-VP2-immunized animals developed significant neutralizing antibody response against BTV-4 (Fig. 3a) and RVFV (Fig. 3b) compared to mock-vaccinated mice; with a mean of 2.21 ± 0.24 and 4.06 ± 0.22 log PRNT_50_ (plaque reduction neutralization test), respectively. The recombinants MVA-GnGc-NS1 and MVA-GnGc-NS1-Nt induced neutralizing antibodies specific of RVFV in the immunized mice (log PRNT_50_ of 3.72 ± 0.31 and 3.73 ± 0.15, respectively; Fig. 3b), but not against BTV-4 as expected^19^ (Fig. 3a).

The presence of specific antibodies to VP2 and NS1 in serum was also analyzed by ELISA at 14 days after boost. Antibodies against VP2 were observed in MVA-GnGc-VP2-vaccinated mice indicating seroconversion (Fig. 3c), with a mean value of 1.79 optical density (OD)_450nm_ (1/50 serum dilution). On the other hand, NS1-specific antibodies were detected in the groups immunized with MVA-GnGc-NS1 and MVA-GnGc-NS1-Nt, with mean levels of 0.62 and 0.34 OD_450nm_, respectively, at the lowest serum dilution tested (Fig. 3d).

**Fig. 4:**
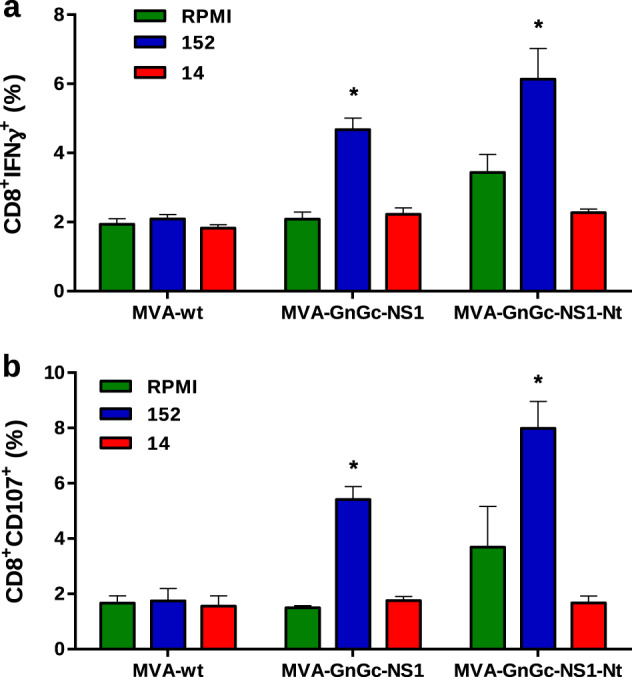
MVA recombinant vaccines in mice elicit cellular immune responses. Percentage of CD8+ IFN-γ+ cells (**a**) and CD8+ CD107+ (**b**) after stimulation with peptides 152 and 14 (irrelevant peptide) or no stimulation (RPMI). The columns represent mean of the group and error bars the SD. The Mann–Whitney *U* test was used for statistical comparisons; *p ≤ 0.05.

In addition to the virus-neutralizing activity induced by either GnGc or VP2 proteins delivered by the recombinant MVAs, we determined, by intracellular cytokine staining, the ability of NS1 and NS1-Nt proteins expressed by the dual MVAs to elicit specific T-cell immune responses. Ten days after the second vaccination with MVA-GnGc-NS1 and MVA-GnGc-NS1-Nt, whole splenocytes from mice (*n* = 4) were restimulated with the NS1-specific T-cell peptide (namely #152), as well as with an irrelevant (off target) peptide (namely #14), for 6 h. The restimulation of spleen cells with the NS1 peptide 152 significantly increased (*p* < 0.05) the expression of IFN-γ and CD107a by CD8+ T cells from MVA-GnGc-NS1 and MVA-GnGc-NS1-Nt-vaccinated mice (Fig. 4). CD107a has been described as a marker of cytotoxic CD8+ T-cell degranulation and cytotoxic activity^30^, and these results indicated that NS1 and NS1-Nt antigens expressed by dual MVA vaccines were capable of inducing a strong CD8+ T-cell response, and activating the induction of CTLs in vivo.

### Protection against BTV in IFNAR^(−/−)^ mice

**Fig. 5:**
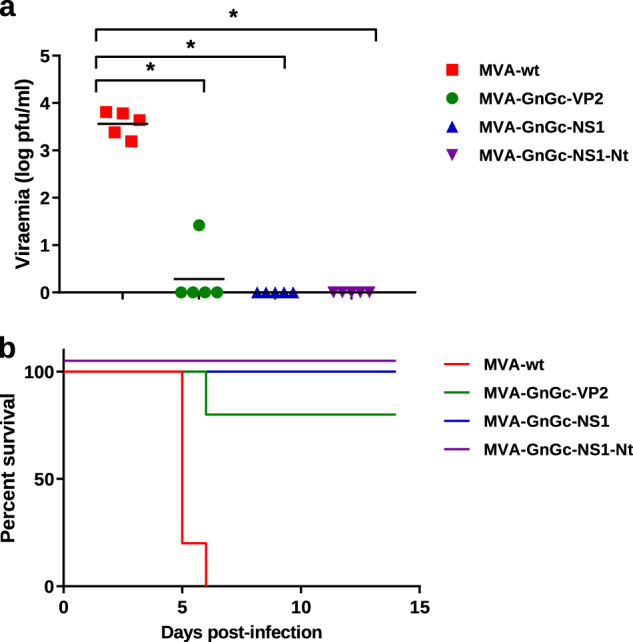
Protection conferred by dual MVA recombinant vaccines against BTV in IFNAR^(−/−)^ mice. **a** Viremia at 5 d.p.i. expressed as log PFU/ml of mice at day 5 after challenge with 500 PFU of BTV-4. Differences between groups were calculated by multiple *t-*test analysis using the Sidak–Bonferroni method. **b** Survival rates of vaccinated and non-vaccinated mice after challenge. Survival data were analyzed using a log-rank test with mice grouped by immunization strategy; *p ≤ 0.05.

We then determined the protective capacity of immunization regimes with MVA-GnGc-NS1, MVA-GnGc-NS1-Nt, and MVA-GnGc-VP2. Groups of IFNAR^(−/−)^ mice (*n* = 5) immunized in a prime-boost regimen at 3-week interval with the recombinant MVAs or with MVA-wt were challenged 2 weeks after the second immunization with a lethal dose of BTV-4 (10^7^ plaque forming units (PFU) per mouse). The non-immunized animals showed clinical signs as early as 3 d.p.i., including ruffled hair, ocular discharges, and reduced activity. These mice were euthanized between 5 and 6 d.p.i. when clinical scores reached humane endpoint. Viremia rapidly increased in all non-vaccinated mice at 3 d.p.i. and reached a mean value of 3.56 log PFU/ml at 5 d.p.i. (Fig. 5a). All mice immunized with MVA-GnGc-VP2 remained healthy, except for one individual, which displayed clinical signs and viremia at 5 d.p.i. and was euthanized (Fig. 5a, b). In contrast, none of the mice vaccinated with two doses of MVA-GnGc-NS1 and MVA-GnGc-NS1-Nt developed clinical signs of disease throughout the experiment. Viremia was not detected at any time point during the experiment in the latter groups of mice (Fig. 5a). Together, these data indicate that immunization with MVA-GnGc-VP2, MVA-GnGc-NS1, or MVA-GnGc-NS1-Nt induces protection in IFNAR^(−/−)^ mice upon a lethal BTV-4 challenge.

### Protection against RVFV in BALB/c mice

**Fig. 6:**
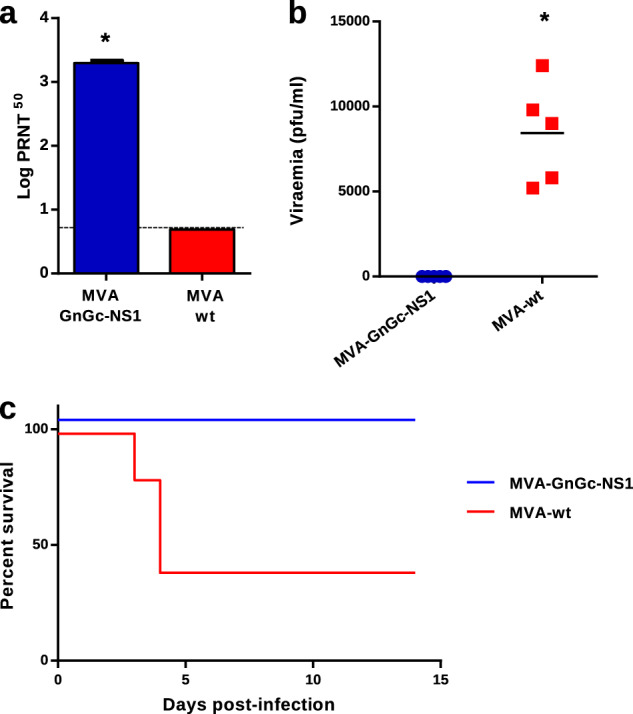
Protective efficacy by dual MVA-GnGc-NS1 immunization after challenge with RVFV. Balb/C mice were inoculated with MVA-wt or MVA-GnGc-NS1, and then infected with 500 PFU of RVFV at day 35. **a** Level of neutralizing antibodies against RVFV at 14 d.p.v. **b** Viremia at 3 d.p.i. measured by plaque assay on Vero cells and expressed as PFU/ml. Horizontal lines indicate mean. Statistical differences were calculated by Mann–Whitney nonparametric tests. **c** Survival rates of vaccinated and non-vaccinated mice after challenge; *p ≤ 0.05.

Previous work in our laboratory has shown that MVA-NS1 protects against multiple serotypes of BTV^19^. Based in our previous results, we chose the best vaccine candidate, MVA-GnGc-NS1, to continue our studies in order to assess its immunogenicity and protective efficacy against RVFV. To analyze the protective immunity elicited upon MVA-GnGc-NS1 immunization, BALB/c mice were used as mouse model for RVFV challenge. Groups of mice (*n* = 5) were inoculated with two doses of MVA-wt or MVA-GnGc-NS1 3 weeks apart. At day 14 post booster, mice were bled for antibody analysis and challenged with a dose of 500 PFU of RVFV 56/74 strain, viremia was assessed at 3 d.p.i. and clinical manifestations were monitored for 2 weeks. MVA-GnGc-NS1 immunization in BALB/c mice resulted in a robust anti-RVFV neutralizing antibody response, similar to that observed in IFNAR^(−/−)^ mice, reaching a log PRNT_50_ titer of 3.29 ± 0.03 (Fig. 6a). At 3 d.p.i., all non-vaccinated mice displayed clinical signs of disease, including ruffled hair, hunching, weight loss, and reduced activity. Three of these mice had to be euthanized following clinical scores reaching the humane endpoint. The remaining two mice recovered by 7 d.p.i. (Fig. 6c). In contrast, all MVA-GnGc-NS1-immunized mice did not develop any clinical signs and were healthy throughout the experiment. Viremia was detected in all non-vaccinated animals at 3 d.p.i., with titers between 5.2 × 10^3^ and 1.2 × 10^4^ PFU/ml, whereas no infectious virus was found in blood of any mice that received MVA-GnGc-NS1 vaccine (Fig. 6b).

### MVA-GnGc-NS1 confers protection in sheep after BTV challenge

**Fig. 7:**
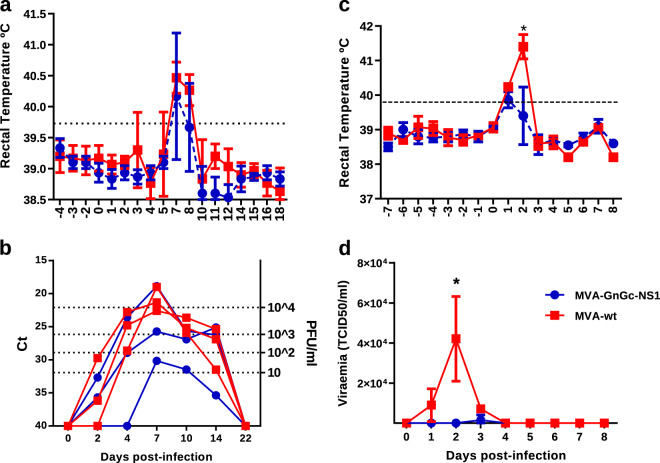
Analysis of protection elicited by MVA-GnGc-NS1 immunization in sheep after infection with BTV or RVFV. **a** Mean rectal temperature responses in MVA-GnGc-NS1-vaccinated and MVA-wt non-vaccinated sheep from −4 to 18 d.p.i. with BTV. **b** Viremia analyzed by real-time RT-PCR from 0 to 22 d.p.i. with BTV. Results are expressed as Ct (left *y*-axis) and PFU equivalents (right *y*-axis and dotted horizontal lines). The real-time RT-qPCR specific for BTV segment 5 was performed as described by Toussaint et al.^69^ and sheep blood containing different known concentrations of virus were used as internal standards, and the Ct values indicated in the left *y*-axis^19^. **c** Mean rectal temperatures in vaccinated and non-vaccinated sheep after RVFV infection. Dotted line represents the fever threshold. **d** Means of viremia analyzed by plaque assay after infection with RVFV. Dots indicate media of the group. Error bars represent SD. Differences between groups were calculated by multiple *t*-test analysis using the Sidak–Bonferroni method; *p ≤ 0.05.

The data on protection against BTV-4 and RVFV conferred by MVA-GnGc-NS1 to mouse models prompted us to test its efficacy as potential vaccine for ruminants. Three sheep were inoculated twice with 10^8^ PFU of either MVA-GnGc-NS1 or MVA-wt at days 0 and 28, and no clinical display nor adverse effects were noticed in any animal. At 31 days post boost (d.p.b.), the sheep were challenged subcutaneously with 10^6^ PFU of BTV-4M strain (isolated from sheep blood in KC insect cells and not previously passed through mammalian cell lines), and viremia and clinical signs analyzed for 3 weeks. At 7 d.p.i., all non-vaccinated sheep developed pyrexia with a mean value of 40.47 °C, whereas vaccinated sheep showed lower temperature (mean 40.16 °C). Moreover, pyrexia in control animals persisted at 8 d.p.i. (mean 40.27 °C), while the mean rectal temperature of vaccinated sheep decreased below the baseline levels (39.6 °C; fever threshold ≥ 39.73 °C, described in “Methods” section; Fig. 7a).

Since the virus present in the blood of sheep infected with BTV-4M does not form lysis plaques in Vero cells, viremia was tested by real-time RT-PCR (Fig. 7b). Viral RNA was detected in non-vaccinated sheep starting from 2 d.p.i., reaching higher levels at 7 d.p.i. (mean Ct 20.9, ≥10^4^ PFU/ml). In contrast, lower levels of viral RNA were detected in MVA-GnGc-NS1-immunized group from 2 to 14 d.p.i. (mean Ct 24.9 and two out of three sheep ≤10^3^ PFU/ml at 7 d.p.i.) compared to control sheep, indicating that the vaccinated sheep partially prevented BTV replication. These results indicate that the immunization of sheep with a homologous prime-boost of MVA-GnGc-NS1 confers partial protection against BTV-4M and reduces viremia and clinical signs.

### MVA-GnGc-NS1 confers protection in sheep after RVFV challenge

Three weeks after BTV infection, animals were completely recovered and were bled to analyze RVFV neutralizing antibodies. Titers of antibodies ranged from 2.23 to 2.37 log PRNT_50_. At 52 d.p.b., sheep were challenged subcutaneously with a dose of 10^7^ PFU of virulent RVFV 56/74 strain and monitored for 8 days.

After infection, all non-vaccinated animals responded with pyrexia at 1 d.p.i. (mean 40.23 °C) and peak temperatures occurred in this group at 2 d.p.i. (mean 41.4 °C). In contrast, vaccinated animals did not increase in mean rectal temperature at 2 d.p.i. (39.4 °C) compared to their mean baseline value at 0 d.p.i. (39.73 °C). Although at 1 d.p.i., vaccinated sheep did slightly surpass (mean 39.8 °C) the fever threshold, they had lower temperatures compared to control group (Fig. 7c).

High levels of viremia were found in non-vaccinated sheep from 1 to 3 d.p.i. (Fig. 7d), with a mean peak value of 4.21 × 10^4^ PFU/ml at 2 d.p.i. In contrast, only one vaccinated sheep (number 982) showed viremia at 3 d.p.i. (4.5 × 10^3^ PFU/ml), but lower than the non-vaccinated sheep and the two remaining sheep did not developed viremia at any time of the experiment.

### Biochemical parameters after RVFV infection

**Fig. 8:**
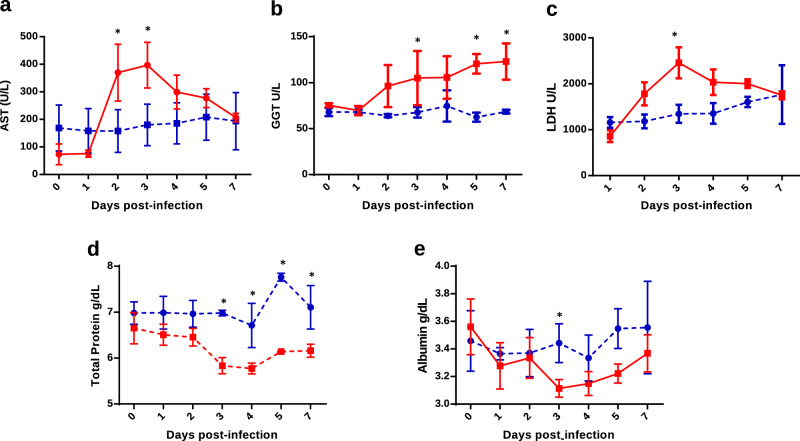
Biochemistry parameters in MVA-GnGc-NS1 and MVA-wt immunized sheep after challenge with RVFV. **a** Aspartate transaminase (AST). **b** Gamma-glutamyltransferase (GGT). **c** Lactate dehydrogenase (LDH). **d** Total protein. **e** Albumin. Error bars represent SD. Statistical differences were calculated by multiple *t*-tests; *p ≤ 0.05.

RVFV infection induces aberrations in biochemistry values, especially in liver enzymes. To confirm the protection elicited by MVA-GnGc-NS1, blood biochemistry parameters were analyzed in immunized and non-immunized sheep after infection with RVFV. The control group responded with increased concentrations of the enzyme aspartate transaminase (AST) from 2 to 7 d.p.i. with peak levels of 470.2 U/l occurring at 3 d.p.i. (normal range = 60–280 U/l). In contrast, MVA-GnGc-NS1-vaccinated sheep maintained their serum AST concentrations at baseline levels throughout the study (Fig. 8a). Moreover, AST concentrations were significantly higher in the control group compared to the vaccinated group at 2 and 3 d.p.i. (*P* < 0.05). Gamma-glutamyltransferase (GGT) serum levels notably increased in the non-vaccinated group from 2 d.p.i., reaching peak levels of 137 U/l. GGT increases were statistically significant at 3, 5, and 7 d.p.i. when compared to vaccinated sheep (Fig. 8b), in which concentrations of GGT remained at baseline levels throughout the study There were also differences in the levels of the enzyme lactate dehydrogenase (LDH) between vaccinated and non-vaccinated sheep after infection with RVFV (Fig. 8c). Non-vaccinated sheep responded to challenge with increased concentrations of LDH, reaching peak levels of 2816 U/l at 3 d.p.i. This elevation in the level of LDH in control sheep was statistically significant compared to vaccinated animals.

Total protein concentration in serum was also analyzed and control animals showed decreased values after infection (Fig. 8d). In non-vaccinated animals, the means of total protein concentrations were significantly lower than in vaccinated sheep from 3 to 7d.p.i. Albumin level in sera from non-vaccinated sheep also decreased after infection with RVFV. In particular, there was a significant decline in albumin levels at 3 d.p.i. compared to vaccinated animals (Fig. 8e). These results are consistent with other studies of viral infections like hepatitis B or HIV that showed a decrease in the serum total protein and albumin level^31,32^. Therefore, the study of biochemical markers showed that sheep vaccinated with MVA-GnGc-NS1 were protected against RVFV infection.

### Histological findings

**Fig. 9:**
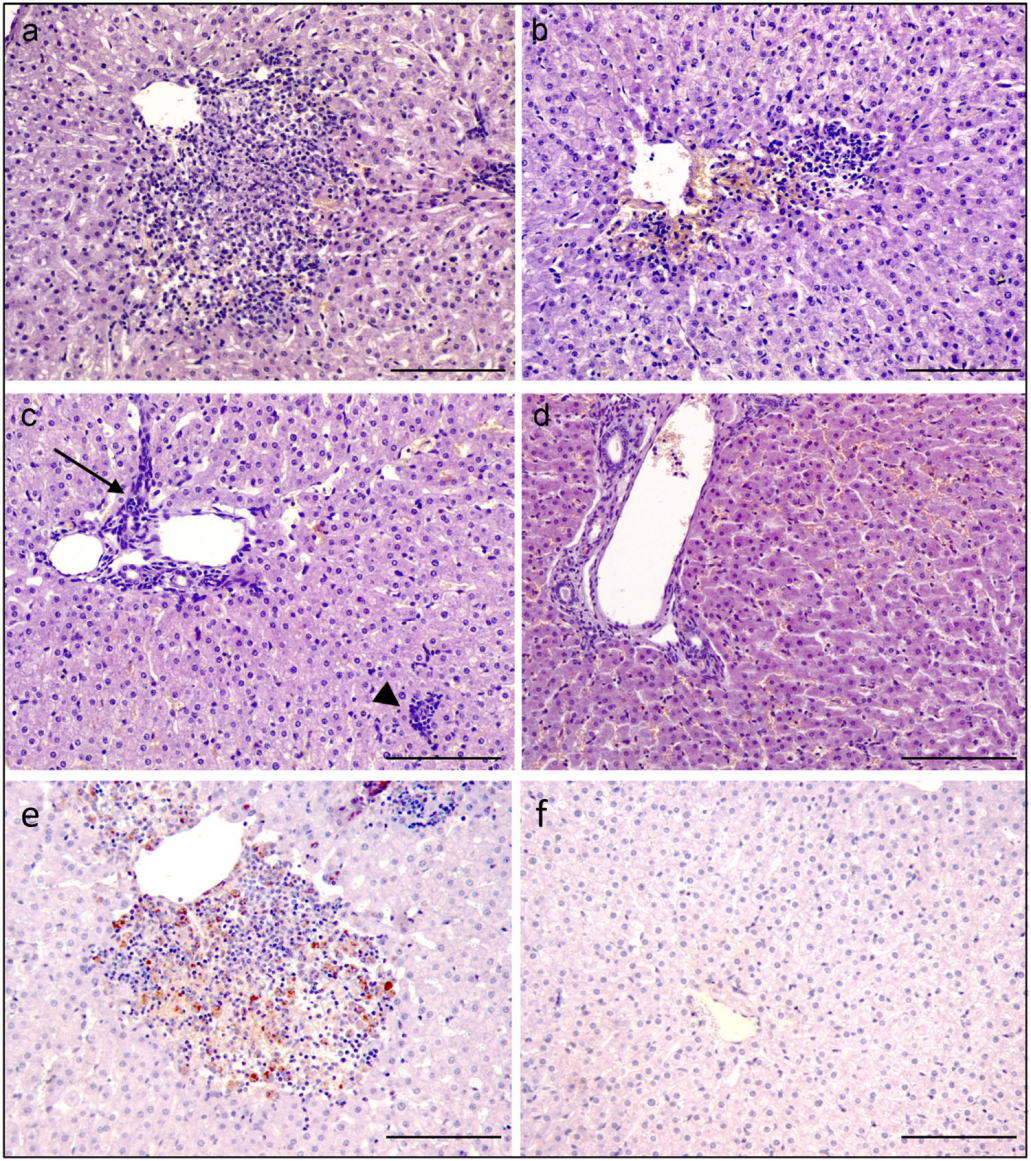
Histological appearance of the liver from non-vaccinated and vaccinated ewes after RVFV challenge. **a** Non-vaccinated sheep at 4 d.p.i. Necrotic hepatitis characterized by a focus of central necrosis, surrounded by a mixed infiltrate of lymphocytes, macrophages, and plasma cells, adjacent to centrolobular vein. **b** Non-vaccinated sheep at 7 d.p.i. Similar, but milder, lesion to the previous sheep. In this case, there is not an evident central area of necrosis and the inflammatory infiltrate is mostly formed by mononuclear cells. **c** Non-vaccinated sheep at 8 d.p.i. Mild non-purulent hepatitis. Increase number of mononuclear cells in the portal area (arrow), and a small aggregate of lymphocytes and macrophages (arrowhead). **d** Vaccinated sheep at 7 d.p.i. No significant hepatic lesions were found in vaccinated animals. **e** Non-vaccinated sheep at 4 d.p.i. Intracellular labeling of RVFV antigen (brown-golden color) within a focus of necrosis and inflammation adjacent to a centrolobular vein. RVFV antigen IHC. **f** Vaccinated sheep at 4 d.p.i. Hepatic lobule with no significant lesions or labeling of viral antigen; RVFV antigen. Magnification 200×. Scale bars 200 µm.

Multifocal necrotizing hepatitis is the most characteristic lesion of RVF cases in adult sheep^33^. To assess the protection conferred by MVA-GnGc-NS1 immunization against RVFV, postmortem samples of the liver from all sheep were evaluated through histological and immunohistochemical methods. Multifocal hepatitis was found in every liver sample from the non-vaccinated RVF-challenged animals. The most severe lesions were found in non-vaccinated sheep euthanized at 4 d.p.i., as there were several foci with central area of necrosis and hemorrhage surrounded by a moderate infiltration of lymphocytes, macrophages, and less numerous, neutrophils (Fig. 9a). The lesions in non-vaccinated ewe culled at 7 d.p.i. were similar but milder, as the foci were smaller and they were mainly composed of inflammatory infiltrate, where the necrosis was mostly absent (Fig. 9b). Finally, non-vaccinated sheep culled at 8 d.p.i. showed mild portal hypercellularity and small and scant aggregates of non-purulent inflammatory cells in the parenchyma (Fig. 9c). Samples from vaccinated sheep only showed mild portal hypercelullarity (Fig. 9d). Immunohistochemical labeling of RVFV antigen showed the presence of the virus in relation to the necrotic and inflammatory foci in the liver from non-vaccinated animals (Fig. 9e), while no detection of viral antigen was observed in samples from vaccinated sheep (Fig. 9f), except for one small isolated foci of infected cells in the vaccinated sheep #982 that displayed mild viremia levels (data not shown). All these data indicate that immunization of sheep with MVA-GnGc-NS1 confers considerable protection against a RVFV challenge, and reduces or abrogate viremia and clinical signs.

## Discussion

RVFV and BTV are two important pathogens that seriously affect ruminants, causing huge losses in livestock. These viruses share several epidemiology aspects, making highly favorable the development of a bivalent vaccine to protect against both viruses. The main hosts of RVFV and BTV are ruminants, and particularly sheep that is severely affected. Periodic vaccination campaigns in Europe are needed to control the spread of BTV, that has become endemic to Southern Europe^34^. However, current BTV vaccines are serotype specific^34^. On the other hand, the vector competence of European mosquitoes indicates that RVF outbreaks in Europe are possible^35^. Over the past decades, diverse recombinant vaccine candidates have been explored to prevent BTV or RVFV separately. However, immunization with multivalent vaccines has potential advantages in reducing costs. In this work, we describe a bivalent vaccination approach that offers high protection against BTV and RVFV simultaneously.

MVA vector vaccines for viral infections have proven to be effective against a wide range of viral diseases^36^. Moreover, the use of MVA deployed as viral vaccine vector induces strong antibody and long-lasting T-cell responses targeting intracellular pathogens^37–39^. In the present study, several recombinant MVAs expressing BTV and RVFV proteins were generated. In vivo studies demonstrated that homologous prime-boost immunizations with MVA-GnGc-VP2-, MVA-GnGc-NS1-, or MVA-GnGc-NS1-Nt-protected mice against a lethal challenge with BTV. MVA-GnGc-VP2 induced high levels of neutralizing antibodies specific of BTV-4. It has been demonstrated that VP2 alone is sufficient to elicit protective immune responses against BTV^14^. In fact, experimental protein-based BTV vaccines used to include the immunodominant VP2 protein, although they are serotype specific^40–42^. MVA-GnGc-NS1- or MVA-GnGc-NS1-Nt-immunized mice did not elicited neutralizing antibodies specific of BTV-4, but developed strong cytotoxic CD8 T-cell responses against NS1 and they were protected against BTV. Previous work in our laboratory showed that CD8 T-cell responses raised against NS1 protein plays a role in cross-protective immunity among BTV serotypes^18,19^. Since the protection against BTV induced by the three rMVAs generated was very similar in mice and that a multiserotype vaccine against BTV is being sought, we decided to test the protection elicited against RVFV by rMVA-GnGc-NS1. Furthermore, vaccination with the three recombinant MVAs also triggered a robust response of RVFV neutralizing antibodies and we did not observe significant differences among the three rMVAs. MVA-GnGc-NS1-immunized mice did not develop any clinical signs or viremia after infection with RVFV and were healthy throughout the experiment. Importantly, the immunization with MVA-GnGc-NS1 conferred protection against a lethal challenge with BTV-4 and RVFV in mice.

Notably, prime-boost immunization with MVA-GnGc-NS1 was able to provide immunity against BTV and RVFV in sheep. Two out of the three animals vaccinated with MVA-GnGc-NS1 showed lower viremia and clinical signs than non-vaccinated animals after challenge with BTV-4M. Although the viremia was not abrogated in the immunized sheep, the titers of virus found in two out of three sheep were 10^3^ PFU/ml or lower. Experimental infections of *Culicoides sonorensis* with BTV-11 and BTV-1 infected blood showed that the efficiency of infection of midges was dose-dependent and the 50% Midge Alimentary Infective Dose (MAID_50_) was roughly calculated to a blood meal titer of ±2 × 10^5^ and ±10^6^ Median Tissue Culture Infectious Dose (TCID_50_)/ml for BTV-11 and BTV-1, respectively^43,44^. According to these experimental infections, the level of virus detected in the blood of the MVA-GnGc-NS1-vaccinated sheep was 200 times lower than the minimal dose required for the insect vector infection, not being sufficient to infect midges and then avoiding the transmission of the virus.

After RVFV infection in sheep, mean rectal temperatures were lower in MVA-GnGc-NS1 vaccinated than in non-vaccinated animals. Moreover, viremia was significantly reduced in vaccinated animals compared to controls. Importantly, no infectious virus was detected in blood from two out of three vaccinated animals throughout the experiment. These results indicate that MVA-GnGc-NS1 immunization elicits immune protection against RVFV. Interestingly, previous works of immunization with a similar rMVA-GnGc vaccine did not show a strong RVFV neutralizing antibody response in mouse or sheep^10,21^ and failed to protect sheep upon two serial immunizations^21^. Although comparative (side by side) experiments might be needed, it is reasonable to speculate that the different source of MVA vector and/or the different locus/promoter used in this work could explain the improved immunogenicity against the encoded RVFV glycoprotein antigens. In this sense, it has been described that genome location and TK function can contribute to the relative immunogenicity of antigens when expressed from rMVA^45^. In addition, in previous rMVA-GnGc vaccine construct^10^, the heterologous gene was cloned under the control of the vaccinia 7.5 k early/late promoter, while in the MVA-GnGc-NS1 describe here the RVFV glycoproteins genes were placed under the control of an optimized strong early/late promoter^46,47^. The different locus and promoters where GnGc was located in the MVA could explain the differences between rMVA-GnGc and MVA-GnGc-NS1 observed in protection against RVFV. Moreover, the slight differences in the encoded sequences (lack of N-terminal tpA signal peptide, or C-terminal marker tags) may also account for the differences in immunity and efficacy observed. In any case, these data clearly shown that our MVA-based vaccine approach remains as a valid resource strategy for further RVF vaccine developments.

Besides vaccinated sheep showed no obvious clinical signs and viremia was reduced or eliminated, the study of markers of liver damage and the pathological analysis confirmed the efficacy of MVA-GnGc-NS1 in protection against RVFV, blocking viral dissemination in secondary tissues. Increased AST, LDH, and GGT enzyme activity in the serum are sensitive markers of liver damage^48–50^. We observed a significant increase of these enzymes in non-immunized sheep after RVFV challenge compared to vaccinated animals. The increase in AST, GGT, and LDH after infection with RVFV was most probably due to the hepatic lesions, as has previously been described^21,51,52^. Consistent with an absence of liver damage, the levels of AST, GGT, and LDH did not significantly change in vaccinated sheep after challenge with RVFV. These results confirm that MVA-GnGc-NS1 immunization protects animals from liver damage caused by RVFV. Finally, when postmortem studies were performed, non-vaccinated and RVFV-challenged sheep showed mild to moderate multifocal necrotic hepatitis and the viral antigen was detected in relation to those lesions. These findings are similar to those lesions found in natural cases of RVFV infection sheep, where necrotizing hepatitis is the hallmark lesion^53,54^. On the other hand, the vaccinated sheep showed no necrosis or aggregation of inflammatory cells in the hepatic parenchyma, where virus was mostly absent in two out of three sheep.

Despite the availability of live-attenuated and inactivated vaccines against RVFV and BTV, both are known to have a number of limitations and safety concerns^55,56^. Several new experimental BTV vaccines are under development and show improvements over classical vaccines as safety, and DIVA (differentiate infected from vaccinated animals) capability, especially those based on poxvirus vectors^19,20,57,58^ and BTV reverse genetic as the Disabled Infectious Single Cycle^59^ or the Disabled Infectious Single Animal vaccines^60,61^. In addition to the demonstrated efficacy of MVA as a vaccine vector for BTV and RVFV individually, we take advantage of its ability to allow the cloning of several genes in its genome to develop a bivalent vaccine against these two viruses. Severe outbreaks of RVFV usually takes place after long inter-epizootic periods with no detectable virus circulation^62^, thus there is a need for safer and efficacious veterinary RVFV vaccines. A bivalent vaccine able to protect against BTV and RVFV, would be useful in Africa and outside, promoting vaccination of ruminants in countries where sporadic RVF disease outbreaks occur. Regarding temperature stability of MVA as a candidate vaccine against BTV and RVFV to be used in Africa, it has been demonstrated that MVA was stable at 37–45 °C for a month. This viral vector could be dried without using membranes and still retains its infectivity when reconstituted. Furthermore, the dried MVA viral vector could be stored for up to 12 months at 37 °C (ref. ^63^).

In conclusion, MVA-based vaccines generated in the present work provide an opportunity to potentially deliver protection against both ruminant infections BTV and RVFV. In addition, the antigenic variability of BTV, with at least 27 different serotypes, is a major concern to control the spread of this virus through vaccination campaigns and the inclusion of NS1 in the vaccine composition can overcome this problem, representing a huge advance over existing vaccines in tackling all serotypes of BTV.

## Methods

### Ethics statement

Animal experimental protocols were approved by the Ethical Review Committee at the INIA-CISA and Comunidad de Madrid (Permit number: PROEX 037/15) in strict accordance with EU guidelines 2010/63/UE about protection of animals used for experimentation, and other scientific purposes and Spanish Animal Welfare Act 32/2007.

### Viruses and cells

Vero cells (ATCC; catalog no. CCL-81), BHK-21 cells (ATCC; catalog no. CCL-10), and chicken embryo fibroblasts (DF-1; ATCC; catalog no. CRL-12203) were grown in Dulbecco’s modified Eagle’s medium (DMEM) supplemented with 2 mM glutamine, 10% heat-inactivated fetal bovine serum (FBS), and antibiotics. Virus stocks were generated by infection of 80% confluent cells using a multiplicity of infection (MOI) of 0.1. RVFV 56/74 and RVFV MP12 viral stocks were performed on BHK-21 cells as previously described^21^. TCID_50_ titers of this stock were determined on Vero cells by the method of Reed and Muench^64^. BTV serotype 4 (SPA2004/02; BTV-4) and BTV serotype 4 strain Morocco (MOR2009/09; BTV-4M) were grown on Vero cells and titrations were performed by plaque assays on Vero cells. Modified vaccinia virus Ankara (MVAΔF13L)^65^ was growth and titered on DF-1 cells.

### Construction of recombinant MVA-GnGc

RVFV MP12 glycoprotein GnGc sequence was amplified by PCR. Plasmid pCMV-M4 (ref. ^22^) was used as a template for PCR amplification using the forward primer 5′-CGGAATTCATGGCAGGGATTGCAATGACAGTCC-3′ (EcoRI underlined), and the reverse primer 5′-CGGGATCCACTGATCTATGAGGCCTTCTTAGTG-3′ (BamHI underlined) and inserted in plasmid pMVA-βGus^65^ previously digested with EcoRI and BamHI enzymes, to generate pMVA-GnGc plasmid. The pMVA-GnGc transfer plasmid contained the F13L gene of MVA and the GnGc gene under the control of a vaccinia virus (VV) early/late promoter. Recombinant MVA-GnGc were generated by infecting DF-1 cells with MVAΔF13L at a MOI of 1 (MOI = 1), and transfecting them with the transfer plasmid pMVA-GnGc. Cell cultures were harvested at 48 h.p.i., and MVA-GnGc was selected and cloned four times by plaque isolation assay.

### Construction of MVA-GnGc-NS1, MVA-GnGc-NS1-Nt, and MVA-GnGc-VP2

The MVA transfer plasmids pSC11-NS1, pSC11-NS1-Nt, and psC11-VP2 containing the NS1, NS1-Nt (N-terminal region of NS1 (1–270 amino acids)), and VP2 BTV-4 genes inserted into the thymidine kinase site of MVA, and under the control of the VV early/late promoter p7.5 were previously generated^19,20^. Recombinant MVA-GnGc-NS1, MVA-GnGc-NS1-Nt and MVA-GnGc-VP2 were prepared by infecting DF-1 cells with MVA-GnGc at a MOI of 0.5 (MOI = 0.5), and transfecting them with the transfer plasmids pSC11-NS1, pSC11-NS1-Nt, or pSC11-VP2, respectively. Cell cultures were harvested at 48 h.p.i., and the dual recombinant MVAs were selected after plaque assay by the addition of X-Gal to the agar overlay. Dual MVAs were cloned four times by plaque isolation assay.

### Confocal microscopy

DF-1 cells were grown on coverslips in M24 plates and infected with the different recombinant MVA viruses. After 48 h.p.i., cells were washed with phosphate-buffered saline (PBS) and fixed with 4% paraformaldehyde for 10 min. The cells were permeabilized by using 0.1% Triton X-100 in PBS for 5 min. Nonspecific reactivity was blocked after incubation of cells with 20% FBS–PBS for an hour. Sheep and mouse antisera raised against BTV-4 (dilution 1:500) and RVFV (dilution 1:500) were incubated overnight at 4 °C. Specific secondary antibodies conjugated to Alexa fluor 488 (cat. number A11015, Thermofisher) and Alexa fluor 596 (cat. number A11032, Thermofisher) were used at 1:1000 dilution for the assays. Nuclei were visualized by using DAPI. Laser scanning confocal microscopy images were acquired with an inverted Zeiss Axiovert LSM 880 microscope. Images were analyzed with Zen 2.0 (Carl Zeiss) and Fiji (NIH) software packages.

### Mice

Type I interferon receptor defective mice (IFNAR^(−/−)^) on a 129 Sv/Ev background, and BALB/c mice were used for the studies. Mice were housed under pathogen-free conditions and allowed to acclimatize to the biosafety level 3 (BSL3) animal facilities at for the Animal Health Research Center (INIA-CISA), Madrid, before use.

### Immunization of mice and challenge

Groups of IFNAR^(−/−)^ mice (*n* = 9) were vaccinated intraperitoneally at 0 and 21 days with 10^7^ PFU of MVA-GnGc-NS1, MVA-GnGc-NS1-Nt, MVA-GnGc-VP2, or MVAΔF13L (MVA-wt). Ten days after second immunization, four animals of each group sacrificed in order to analyze specific cellular immune responses. Two weeks after second immunization, five animals of each group were bled to determine antibodies against BTV and RVFV, and were challenged subcutaneously with 5 × 10^2^ PFU of BTV-4.

BALB/c mice (*n* = 5) were vaccinated intraperitoneally with two doses (3 weeks apart) of 10^7^ PFU of MVA-GnGc-NS1. Two weeks after second immunization, animals were bled to determine antibodies and were challenged intraperitoneally with 5 × 10^2^ PFU of RVFV.

After infection, animals were evaluated and scored for individual clinical signs. Rough hair (absent = 0, slightly = 1, markedly = 2), activity (normal = 0, slightly reduced = 1, reduced = 2, severely reduced = 3), eye swelling (absent = 0, slightly = 1, moderate = 2, severe = 3), and temperature (normal = 0, hypothermia = 3). The final score was the addition of each individual score. The minimum score was 0 for healthy and 1–11 depending upon the severity. Animals that reached 8 points of score were euthanized. Each score represents the value of a single animal.

### Plaque reduction neutralization test

Twofold dilutions (from 1:5) of heat-inactivated sera (56 °C for 30 min) were incubated with 100 PFU of BTV-4 or RVFV MP12 strain for 1 h at 37 °C. Then, samples were inoculated into 12-well plates containing semi-confluent monolayers of Vero cells. Following incubation for 1 h, an agar overlay (DMEM, 10% FBS, 2% carboxymethylcellulose) was added and plates incubated for 5 (BTV) or 3 (RVFV) days at 37 °C in 5% CO_2_. Plaques were fixed with 10% formaldehyde and visualized with 2% crystal violet PBS. PRNT_50_ titer was calculated as the reciprocal (log 10) of the highest dilution of serum that neutralized 50% of the control virus input. The cutoff is 0.69, log of the reciprocal of the first dilution 1:5.

### Enzyme-linked immunosorbent assays

Indirect enzyme-linked immunosorbent assays (ELISA) were used to determine specific VP2 and NS1 antibody levels before infection^66,67^. MaxiSorp plates (Nunc, USA) were coated with 300 ng/well of purified recombinant VP2 or NS1 protein expressed in Bac-To-Bac Baculovirus expression System (Invitrogen)^20,66^. Mice sera collected before challenge was diluted and analyzed in duplicates. Plates were incubated with an anti-mouse IgG-HRP secondary antibody (cat. number P0447, Dako) at a 1:2000 dilution and the reaction was developed with substrate solution TMB (Sigma) and stopped by adding 50 ml of 3 N H_2_SO. Results were expressed as ODs measured at 450 nm.

### Flow cytometric analysis

For the intracellular cytokine staining assay, 10^6^ splenocytes were stimulated with 10 µg/ml of NS1-152 peptide, 4 µg/ml of concanavalin A as a nonspecific stimulus, 10 µg/ml of peptide 14 as an irrelevant peptide, or left untreated in RPMI 1640 supplemented with 10% FCS. Six hours before the assay, CD107a/LAMP-1-FITC antibody at 1:10 dilution (Clone H4A3 mouse, cat. number 130-102-191, Miltenyi) and brefeldin A (5 µg/ml) were added. After 18 h of stimulation, cells were washed, stained for the surface marker with anti-mouse CD8 PerCP-Vio700 (Clone 53-6.7 mouse, cat. number 130-120-756, Miltenyi) at 1.10 dilution, fixed and permeabilized with PBS 1% FBS, 4% formaldehyde, 1% saponin buffer. Then cells were stained intracellularly using IFN-γ-PE antibody (Clone AN.18.17.24, cat. number 130-102-388, Miltenyi) at 1:10 dilution. Data were acquired by fluorescence-activated cell sorter (FACS) analysis on a FACSCalibur (Becton Dickinson). Gating strategies used to identify CD8+ T-cell populations are showed in the Supplementary Fig. 1. Analyses of the data were performed using FlowJo™ v10.0.8 (Tree Star, Ashland, OR).

### Immunization of sheep and challenge

Six naive healthy sheep (Spanish Churra sheep breed), aged 2 years were acclimated for 7 days at the BSL3 animal facility of the Animal Health Research Center (INIA-CISA) before starting the experiment. All sheep were negative to BTV-4 and RVFV. Animals were subcutaneously inoculated with 10^8^ PFU of MVA-GnGc-NS1 (sheep number 938, 964 and 982) or MVA-wt (sheep number 9, 12, and 69) at days 0 and 28 of the experiment. At 22 days post booster (d.p.b.), prechallenge blood samples were collected from all animals. At 31 d.p.b., sheep were challenged subcutaneously with 10^6^ PFU of BTV-4M. After challenge, all sheep were monitored daily for clinical signs and rectal temperature. Blood samples for virological analyses were collected from days 0 to 7 postinfection (d.p.i.). Animals were allowed to recover for 3 weeks before starting the next experiment. At 52 d.p.b., all sheep were infected subcutaneously with 10^7^ PFU of RVFV. Post challenge, all sheep were monitored daily for clinical signs and rectal temperature. The fever threshold was set to ≥39.73 °C based on the mean plus three standard deviations of the rectal temperatures recorded in six sheeps for 1 week before challenge. Blood samples for virological and blood chemistry analyses were collected from days 0 to 7 d.p.i. At different time points, animals were euthanized, necropsies were performed, and liver samples were collected for histological studies. Sheep 12 and 982 were euthanized at 4 d.p.i., sheep 69 and 964 at 7 d.p.i., and sheep 9 and 938 at 8 d.p.i.

### Viremia analyses

Whole blood samples in EDTA were collected at different times postinfection from challenged mice. For determination of viral titers, 100 µl of blood was washed with PBS, centrifuged, and lysed with 900 µl of distillated sterile water. After 2 min, 100 µl of PBS 10× was added to samples. The amount of infectious virus was measured by standard plaque assay^68^ or TCID_50_ (ref. ^21^) on Vero cells. The real-time RT-qPCR specific for BTV segment 5 was performed as described by Toussaint et al.^69^, and sheep blood containing different concentrations of virus were titrated and used as internal standards of the experiment^19^.

### Biochemistry assays

Whole blood samples were collected in BD Vacutainer tubes and centrifuged at 1267 × *g* for 10 min for serum separation. The serum samples were stored at −80 °C until use and then analyzed for alanine aminotransferase, aspartate aminotransferase (AST), alkaline phosphatase, total protein, and albumin levels using specific reagents according to the manufacturer’s instructions (SpinReact, Vall D’En Bas, Spain). Parameters were selected based on their potential role in liver disease and infection and were measured in a Saturno 100 analyzer (Crony Instruments, Rome, Italy).

### Histopathological and immunohistochemical analyses

Samples from sheep livers were fixed in 10% buffered formalin (pH 7.2). After fixation, samples were dehydrated through a graded series of alcohol to xylol and embedded in paraffin wax. Sections of 3 µm thick from paraffin wax blocks were cut, and stained with hematoxylin and eosin for histopathological analyses.

For immunohistochemical procedures, tissue sections were subjected to microwave treatment (30 min) in citrate buffer (pH 9.0) for antigen retrieval. The endogenous peroxidase activity was inhibited by incubating sections with peroxidase blocking reagent (Dako) for 1 h. After incubation with mouse polyclonal serum against RVFV, sections were incubated for 30 min with the secondary antibody anti-mouse-HRP (cat. number P0447, Dako) at a 1:200 dilution. Peroxidase reaction was developed using 3.3′-diaminobenzidine tetrahydrochloride as chromogen diluted 1:50 in a specific buffer (Dako). Finally, sections were counterstained with hematoxylin, dehydrated, and coverslipped with DePex mounting medium.

### Statistical analyses

Statistical analyses were carried out using GraphPad PRISM version 6.00 (GraphPad Software, San Diego, CA). Differences in antibody levels, T-cell responses, serum biochemical parameters, and viremia between groups were calculated by Mann–Whitney nonparametric or multiple *t*-test analysis. Survival data were analyzed using a log-rank test with mice grouped by immunization strategy. A significance level of *p* < 0.05 was used in all analyses.

### Reporting summary

Further information on research design is available in the Nature Research Reporting Summary linked to this article.

## Supplementary information

Reporting Summary

Supplementary Information

## Data Availability

All data generated or analyzed during this study are included in the main text and Supplementary Information. All relevant data are also available upon request from the corresponding authors.
