# Supplementary material for: A protective bivalent vaccine against Rift Valley fever and bluetongue
Source: NPJ Vaccines. 2020 Jul 30;5:70. doi: 10.1038/s41541-020-00218-y (PMC7393076; doi:10.1038/s41541-020-00218-y)
Supplement: Supplementary file 2 — Supplementary Information [file 41541_2020_218_MOESM2_ESM.pdf]

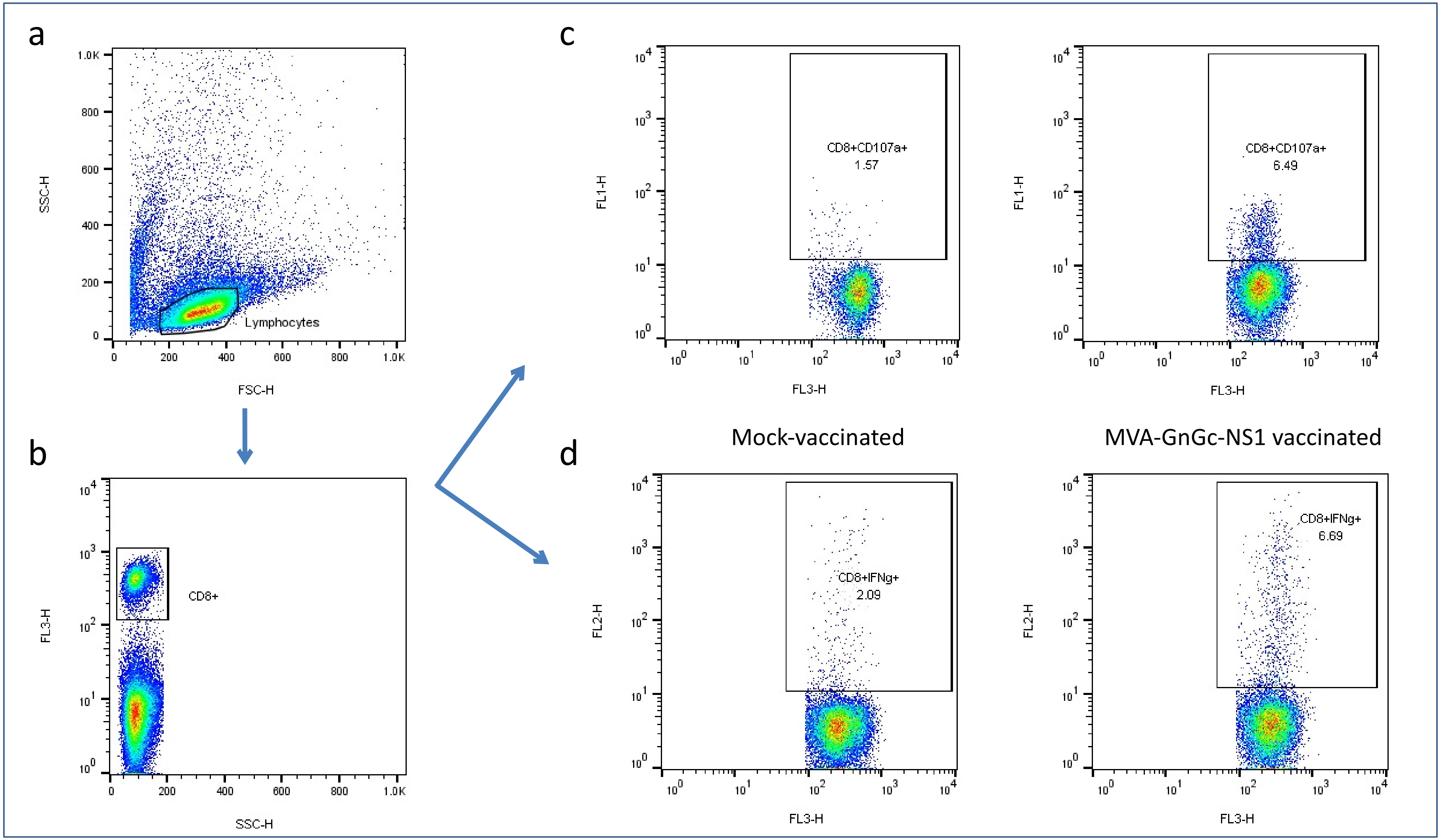

Supplemental Figure 1. Gating strategies used to identify CD8+ T cell populations. Splenocytes from immunized (MVA-GnGc-NS1 and MVA-GnGc-NS1-Nt) and mock-immunized mice were stimulated with peptide 152(NS1). After polycromatic staining of cells with different fluorochrome-conjugated antibodies, flow cytometric analyses were carried out. Lymphocytes were first gated using FSC and SSC (**a**) and then gated on the CD8+ cell population (**b**) using CD8-PerCp-Vio700 antibody. Examples of flow cytometric analyses for CD107a degranulation marker (**c**) and IFN-γ production(**d**) in CD8+ T cell subsets using CD107a-FITC and IFN-γ-PE antibodies in a mock-vaccinated (MVA-wt) mouse or a MVA-GnGc-NS1 vaccinated mouse. Results of the analyses of each group of mice are presented in figure 4 a, b of the manuscript.
